# Spectral-Topological Superefficient Quantum Memory

**DOI:** 10.1038/s41598-018-38244-5

**Published:** 2019-02-07

**Authors:** N. S. Perminov, S. A. Moiseev

**Affiliations:** Kazan Quantum Center, Kazan National Research Technical University n.a. A.N.Tupolev-KAI, 10 K. Marx, Kazan, 420111 Russia

## Abstract

In this work, we propose a universal (spectral-topological) approach towards the realization of the quantum memory, consisting of a small number of controlled absorbers, providing a super-high quantum efficiency of more than 99.9% required for practical quantum information science. In this way, we have found a series of spectral-topological matching conditions for the spectroscopic parameters of the absorbers which ensure the maximal efficiency in the broadband spectral range due to controlling the relative position (topology) of the eigenfrequencies in the absorbers spectrum. We also discuss the implementation of the proposed approach using the modern microwave and optical technologies.

## Introduction

The development of the optical quantum memory (QM) is of decisive importance for quantum information technologies^1–4^. Impressive experimental results on the way to create the efficient QM were achieved in the last decade^5–7^. At the same time, the further improvement of the quantum efficiency (QE) to the values extremely close to 100% remains a complicated unsolved problem. In addition to a number of related tasks, first of all the solution of this problem requires the creation of the high performance quantum interface for the reversible storage of photons in long-lived coherent systems.

One of the promising approaches for creating multimode QM is based on the reversible photon echo on the resonant ensembles in free space^8,9^ and high-Q resonators^10–14^. Owing to the enhancement of the interaction between the resonance system of atoms and light, it was possible to increase considerably the QE and decrease the working number of atoms as it was firstly demonstrated in works^12,15^. Herein, the increase of the QE in a wider spectral range is possible by providing the additional spectral matching conditions^11,16,17^. The general solution of this problem remains unknown that strongly hampers the search for practical ways of creating the high performance broadband multi-qubit QM.

In this work, based on the multiresonator QM^18,19^, we show that a system of a small number of resonant absorbers (quantum dots, artificial atoms, miniresonators etc.) makes it possible to implement the super-high spectral quantum efficiency and fidelity (QEF) larger than 99.9% in the working frequency band. We found that so high QEF could be realized in the vicinity of the parameters, where a topological restructuring of the system spectrum and a change in the number of observed resonance lines are recorded. In comparison with the well-known quantum storage techniques in continuous media, our scheme does not require complex preparation of the storage media^9^ and only needs adjustment of a small number of controlled parameters. In contrast to the previous works^10,19,20^, where only one parameter is optimized, in this work we solve the problem of optimizing all the available parameters of QM by using the spectral-topological (ST) matching condition. The possible experimental implementations of the predicted super-high QEF are discussed for the optical and microwave schemes with realistic experimental parameters.

## Results

### Cascade QM with controllable spectrum

**Figure 1:**
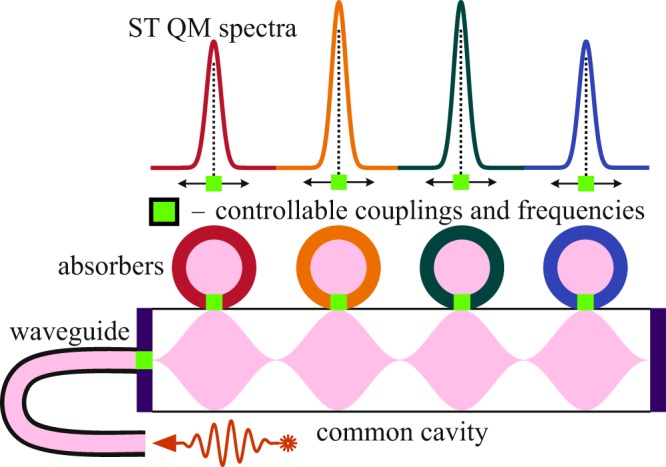
Principle cascade scheme of ST QM: absorbers are connected to the external waveguide through a common cavity with the ability to control the couplings and frequencies of the absorbers.

The general theoretical concept corresponds to the so-called impedance matching photon echo QM in a single mode cavity^10,11,16,21^, which was further extended to ring resonator systems connected with the nanofiber^22^ and other schemes^17–19,23^. The principle cascade scheme of ST QM (Fig. 1) with total control of spectral characteristics consists of several absorbers (microresonators) connected with a common broadband cavity, which is coupled to an external waveguide, where one can also control both coupling with the absorbers and its frequencies. We assume that the absorbers are characterized by a discrete system of narrow resonant lines. Herein, we generalize the realization of so-called AFC-protocol^10,20^, where the resonant atomic frequencies constitute a periodic structure with spectral period ${\rm{\Delta }}$. In this case, the excited atomic coherence leads to the echo pulse emission due to automatic atoms rephasing with time delay $\tau =2\pi /{\rm{\Delta }}$ after the entrance of an input signal field.

Using the input-output formalism of quantum optics^24^ for the studied system, we obtain the equations for the excited modes of absorbers *s*_*n*_(*t*) and common cavity field *a*(*t*):

1 $$[{\partial }_{t}+i{{\rm{\Delta }}}_{n}+{\gamma }_{n}]{s}_{n}(t)+{[{g}_{n}^{0}]}^{\ast }a(t)-\delta {F}_{n}(t)=0,$$

$$[{\partial }_{t}+\kappa /2]a(t)-{\sum }_{n}\,{g}_{n}^{0}{s}_{n}(t)=\sqrt{\kappa }{a}_{in}(t),$$

where ${a}_{in}(t)={[2\pi ]}^{-1/2}\,\int \,d\nu {e}^{-i\nu t}{f}_{\nu }$ is the input pulse, *f*_*ν*_ is the spectral profile of the input pulse, for which the normalization condition for the single-photon field is fulfilled $\int \,d\nu |{f}_{\nu }{|}^{2}=1$, *ν* is the frequency counted from the central frequency of the radiation ${\omega }_{0}$, ${{\rm{\Delta }}}_{n}$ is the frequency detuning of *n*–th absorber, $n\in \{\,-\,N,\ldots ,N\}\backslash \{0\}$, *γ*_*n*_ is the attenuation decrement (decay constant) of the *n*-th absorber coherence and Langevin force *δF*_*n*_(*t*) associated with the relaxation^25^, *κ* is the coupling coefficient of the external waveguide with the common cavity mode, ${g}_{n}^{0}$ is the coupling constant of the common mode and *n*-th absorber. Below we ignore the Langevin forces *δF*_*n*_(*t*) in Eq. (1) by focusing only to the searching of QEF in the studied scheme and we obtain the output field ${a}_{out}(t)=\sqrt{\kappa }a(t)-{a}_{in}(t)$ in terms of the transfer function (TF)^26^ $S(\nu )={\tilde{a}}_{out}(\nu )/{\tilde{a}}_{in}(\nu )$ where

2 $$S(\nu )=(1+iP(\nu ))/(1-iP(\nu )),$$

$P(\nu )=2\nu /\kappa +{\sum }_{n}\,{g}_{n}/({{\rm{\Delta }}}_{n}-i{\gamma }_{n}-\nu )$, ${a}_{in,out}(t)={[2\pi ]}^{-1/2}\,\int \,d\nu {e}^{-i\nu t}{\tilde{a}}_{in,out}(\nu )$, $2|{g}_{n}^{0}{|}^{2}/\kappa ={g}_{n}$ is the effective line width of a separate absorber inside the common broadband resonator (for *γ*_*n*_ = 0). The TF completely determines all the spectral characteristics of the system and its eigenfrequencies are the poles of TF. In the general case TF (2) has a complicated spectral property due to the strong interaction of the absorbers in the common cavity. However, we show that TF can provide a nearly ideal QM under certain condition. In the next part, for the optimization procedure, we will use the approximation *γ*_*n*_ = 0, which has a negligible effect on the parameters for ${\gamma }_{n}\ll 1$.

### Spectral-topological matching conditions

Introducing the spectral delay time $T(\nu )=-\,i\,{\rm{Arg}}(S(\nu ))/\nu$ on frequency *ν*, we can rewrite transfer function (TF) in the form $S(\nu )=|S(\nu )|{e}^{i\nu T(\nu )}$, which is a natural characteristic of the studied linear device in the theory of filters^26^. From here we can formulate the principle for obtaining the high performance broadband QM: delay time (rephasing time) is the same for all frequencies in the given spectral range ${\rm{\Omega }}$, i.e.,

3 $$T(\nu )\cong T({\nu }_{0}),$$

which provides perfect rephasing of all the spectral components for any input light field, where *ν*_0_ is the central frequency of the given range ${\rm{\Omega }}$ (below we assume *ν*_0_ = 0). In particular $|S(\nu )|=1$ and $T(\nu )=T(0)$ for an ideal AFC protocol characterized by the fixed storage time *T*(0).

It was found earlier that the condition (3) can be fulfilled with the accuracy to terms ~*ν*^4^ ^11,16^ and ~*ν*^6^ ^17^ in the vicinity $\nu =0$ that limits anyway the spectral range of the high QE. Below we show that the high QE can be obtained in a wider spectral range by the fulfillment of the equality (3) with higher accuracy. Imposing the larger number of conditions on physical parameters of the system (a set $\{{g}_{n},{{\rm{\Delta }}}_{n}\}$), by using the Taylor decomposition of *T*(*ν*), we consider (3) as an equality in series $T(\nu )-T(0)\cong \sum \,{H}_{\alpha }{\nu }^{\alpha }\to 0$:

4 $$\begin{array}{rcl}{H}_{\alpha } & = & {\partial }_{\nu }^{\alpha }(T(\nu )-T(0)){|}_{\nu =0},\\ {\rho }_{q}({H}_{\alpha }) & = & \sum _{\alpha =1}^{q}\,{H}_{\alpha }^{2}\to \,{\rm{\min }}\,,\end{array}$$

where ${\rho }_{q}({H}_{\alpha })$ is the discrepancy function, $\alpha \in \{0,\ldots ,4N-1\}$ is determined by the maximal number of free parameters of the system. Thus, the fulfillment of the requirement (4) provides high accuracy of equality (3) for the widest possible spectral range.

In analytical calculations we consider the case of small intrinsic losses of resonant absorbers (for example high-Q mini-resonators) under the assumption of the fulfilment of the regime “broadband cavity”, when $N\langle {{\rm{\Delta }}}_{n+1}-{{\rm{\Delta }}}_{n}\rangle /\kappa \le {\gamma }_{n}/\langle {{\rm{\Delta }}}_{n+1}-{{\rm{\Delta }}}_{n}\rangle \ll 1$. With allowance for the spectral symmetry of QM ($T(\nu )=T(\,-\,\nu )$), we find the conditions ${g}_{-n}={g}_{n},\,{{\rm{\Delta }}}_{-n}=-\,{{\rm{\Delta }}}_{n}$ which facilitate the creation of high QE in the broad frequency band. The numerical simulations confirmed this property of the QM, although the analytical proof of necessity condition of the spectral symmetry for maximum QE requires additional studies. By using (2) with ${\gamma }_{n}=0$ in *T*(*ν*) in the direct analytical calculation of Eq. (4), we find the following algebraic system of 2*N* spectral-topological matching conditions on the parameters $\{{g}_{n},{{\rm{\Delta }}}_{n}\}$:

5 $$\begin{array}{rcl}{\rho }_{q}({H}_{2m+1}) & \to  & {\rm{\min }}\,,\\ \frac{{H}_{2m+1}}{(2m+1)!} & = & |\sum _{n=1}^{N}\,\frac{{g}_{n}}{{{\rm{\Delta }}}_{n}^{2m+2}}-\frac{({2}^{2m+2}-1)|{B}_{2m+2}|}{(2m+2)!{[T(0)]}^{-2m-1}}|,\\ T(0) & = & 4\,\sum _{n=1}^{N}\,\frac{{g}_{n}}{{{\rm{\Delta }}}_{n}^{2}},\end{array}$$

where $m\in \{1,\ldots ,2N-1\}$, ${H}_{2m+1}={|{\partial }_{\nu }^{2m+1}(\frac{1}{2}{\rm{tg}}[\frac{\nu T(\nu )}{2}]-\frac{1}{2}{\rm{tg}}[\frac{\nu T(0)}{2}])|}_{\nu =0}$ (which is equivalent to (4)) and *B*_*m*_ are Bernoulli numbers (${B}_{0}=1,\,{B}_{2}=1/6,\ldots$). For optimization in a relatively broad frequency band, we assume that *q* in ${\rho }_{q}$ is equal to the number of free parameters, and for optimization in a fairly wide frequency band, we put $q=2N-1$, which leads us to the spectrally flat function *T*(*ν*) in the central region of the frequency interval and on its borders.

In fact, the conditions (5) are the statement of the problem of the multiparametric optimal control of spectral properties of the QM written in the algebraic form that makes it possible to apply algebraic geometry^27–29^ to search for the ways to improve the QM. In addition, the ST matching conditions (5) can be rewritten through the spectrum {*E*_*n*_} (eigenfrequencies of the system) and can be considered as the conditions for optimizing the spectrum of TF^30^. Below we show that the conditions of the implementation of highly performance broadband QM (3) are associated with the change of the topology of its spectrum and are fulfilled near the point of the spectral-topological transition.

### Topological transitions in the QM spectrum

For the case of the 2*N*-particle system, when the initial frequency modes are detuned equidistantly ${{\rm{\Delta }}}_{\pm n}={\rm{\Delta }}(\,\pm \,n\mp 1/2)$ (further ${\rm{\Delta }}=1$, i.e., the consideration is performed in units of ${\rm{\Delta }}$), and the line widths of modes are the same *g*_*n*_ = *g*, we find $P(\nu )=2g\nu \,{\sum }_{n=1}^{N}\,{[{(n-1/2)}^{2}-{\nu }^{2}]}^{-1}$. Here there is only one free parameter *g* and, for simplicity, we demonstrate the optimization method in a relatively broad frequency band: we put $\rho ={\rho }_{1}$ that leads to single equation for *g*_*cr*_ (such optimization leads to the previously studied matching conditions^10,11^). From (5) for this case and arbitrary *N*, we obtain the following exact relationships for the optimal quantity *g* = *g*_*cr*_ and the time *T*(0) of the signal recovery:

6 $$\begin{array}{rcl}{g}_{cr} & = & \frac{{\rm{\Delta }}}{\pi }{[1-\frac{{\psi }^{(3)}(N+\frac{1}{2})}{{\pi }^{4}}]}^{\frac{1}{2}}{[1-\frac{2{\psi }^{(1)}(N+\frac{1}{2})}{{\pi }^{2}}]}^{-\frac{3}{2}},\\ T(0) & = & \frac{2\pi }{{\rm{\Delta }}}\times \frac{\pi {g}_{cr}}{{\rm{\Delta }}}\times [1-\frac{2{\psi }^{(1)}(N+\frac{1}{2})}{{\pi }^{2}}],\end{array}$$

where ${\psi }^{(m)}(x)$ is the polygamma function. Expanding (6) over $\frac{1}{N}$ we have $\frac{\pi {g}_{cr}}{{\rm{\Delta }}}\simeq 1+\frac{3}{{\pi }^{2}N}$ and $T(0)\simeq \frac{2{\pi }^{2}{g}_{cr}}{{{\rm{\Delta }}}^{2}}(1-\frac{2}{{\pi }^{2}N})$. We see that for optimal QM (at *g* = *g*_*cr*_), the difference in time of the echo signal emission $T(0)=\frac{2\pi }{{\rm{\Delta }}}(1+\frac{1}{{\pi }^{2}N})$ with the case of the absorbers containing an a quite large number of frequencies $T(0)=\frac{2\pi }{{\rm{\Delta }}}$ (for $N\gtrsim 10$)^10,20^ is negligible. But at lower number of the absorbers (*N* < 10) this difference becomes essential. From (6) for *N* = 2 we obtain the following set of spectroscopic data $\{{{\rm{\Delta }}}_{\pm 1}=\pm \,0.5,\,{{\rm{\Delta }}}_{\pm 2}=\pm \,1.5,\,{g}_{\pm 1}\cong 0.37,\,{g}_{\pm 2}\cong 0.37\}$ which corresponds to the efficient storage of narrowband signal obtained in the numerical calculation.

**Figure 2:**
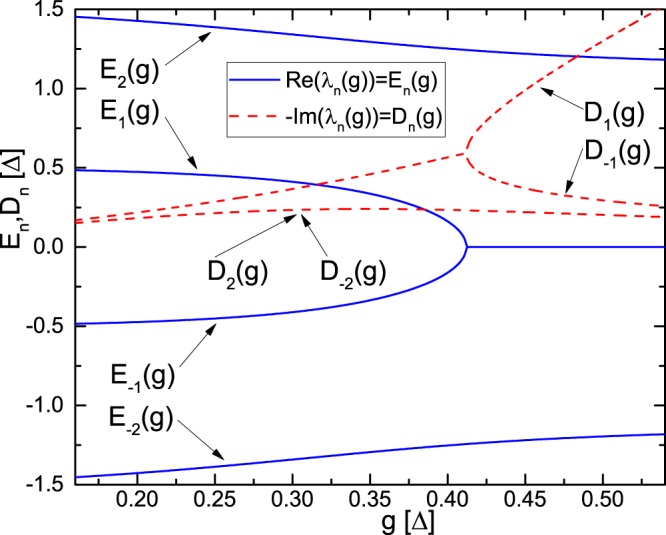
Position of lines ${E}_{n}(g),{D}_{n}(g)$ (in units ${\rm{\Delta }}$) of the TF spectrum of the four-particle system (blue solid and red dashed lines) as a function of the coupling constant *g* (in units ${\rm{\Delta }}$). In the simulation, we assume ${\rm{\Delta }}=1$ without loss of generality, so all the quantities on the figure are dimensionless.

We observed the effect of line merging which reveals the spectral-topological transition in the studied system in the region of the parameters (*g*, ${{\rm{\Delta }}}_{n}$) for which the conditions for achieving a high spectral quantum efficiency are obtained. Physically, the line merging effect demonstrates a sufficiently strong coupling of the absorbers with the common cavity mode which provides large spectral shifts and merging of the original resonant lines. The numerical analysis of the eigenfrequency modes (2) carried out for the broadband common cavity mode (${\rm{\Delta }}/\kappa \ll 1$) and weak relaxation ($\gamma /{\rm{\Delta }}\ll 1$) shows the curve $T(v)/T(0)=1+{A}_{2}{\nu }^{2}+O({\nu }^{3})$ for the case *g* = 0.37 (from (6)). The spectral behavior of *T*(*v*)/*T*(0) characterizes the accuracy of condition (3) near $\nu =0$. Herein, the curve is smoother than for the case $g=0.41$ (i.e. for the line merging of eigenfrequencies in Fig. 2): ${A}_{2}(g=0.37)=0.05$, ${A}_{2}(g=0.37)=-\,0.78$ and $|{A}_{2}(g=0.37)| < |{A}_{2}(g=0.41)|$. It is also seen that the maximal QEF ($g={g}_{cr}$) occurs near the point of the ST transition in the TF spectrum. Thus the TF spectrum of resonant lines consists of 2*N* lines at rather weak coupling constant $g < {g}_{cr}$, while the number of lines decreases by unity, i.e., it is 2*N* − 1 at the coupling strength $g\ge {g}_{cr}$. The ST matching conditions (5) can be also rewritten through the spectrum {*E*_*n*_} and it can be considered as the conditions for optimizing the spectrum of TF.

The ST restructuring in the parametric space occurs in the rather small region of the variation of parameters and is the universal condition of the high quality QEF implementation irrespective of the certain form of the input signal field. For large *N*, the point of maximum QEF and the point of line merging coincide with each other (condition ${{\rm{Discriminant}}}_{\nu }[1-iP(\nu )]=0\Rightarrow {g}_{merg}(N=\infty )=\pi /{\rm{\Delta }}$ (for calculations see^29,31^) and ${g}_{cr}(N=\infty )=\pi /{\rm{\Delta }}$, see (6)). Moreover, by using Eq. (2) we get after algebraic calculations the following form for TF: $S(\nu )=\exp (2i\,{\sum }_{n}\,{\rm{arctg}}[(\nu -{E}_{n})/{D}_{n}])$ (${\gamma }_{n}=0$). Broadband high QEF of the signal pulse retrieval (i.e. $S(\nu )\cong \exp \{i\nu {T}_{0}\}$) is achieved only if the dispersion parameters *D*_*n*_ are close to each other which takes place only for $g\lesssim {g}_{merg}$. Thus, the merging point indicates an area of the optimal parameters where the high QEF is achievable.

Poles of TF and the effect of line merging are also widely used in the signal processing^32^ and in the theory of filters which deals with spectral efficiency improvement^30^. The finer spectral optimization of the QEF can depend on the used frequency band and the form of signal pulses, which, however, requires an additional study taking into account certain parameters of light fields analogous in meaning to that used in the QM scheme based on slow light^33^.

### Optimization of the efficiency in the wide frequency band

To study the properties of the QM in the wide spectral interval irrespective to the form of the signal, we introduce the function of TF spectral errors (cost function analogous^30^) $\delta {S}^{2}(\nu )=|S{(\nu )}^{2}-{S}_{0}{(\nu )}^{2}|$ showing the deviation of TF from the TF of an ideal broadband memory ${S}_{0}(\nu )={e}^{i\nu T(0)}{|}_{{\gamma }_{n}=0}$. The physical meaning of *δS*^2^(*ν*) for small ${\gamma }_{n}\ll 1$ is the energy loss during the storage per unit of frequency ($\delta {S}^{2}(0)\cong 1-\eta (0)$ where $\eta (\nu )=|S(\nu ){|}^{2}$). For optimization in a wide frequency band, we can assume that $\rho ={\rho }_{2N-1}$ which leads us to a smoother function *T*(*ν*) in the central region of the frequency interval, and on its borders. Further in the simulation, we assume ${\rm{\Delta }}=1$ without loss of generality, that is, subsequent calculations are performed in units of ${\rm{\Delta }}$. For *N* = 2 when the initial frequencies of particles are detuned equidistantly ${{\rm{\Delta }}}_{\pm n}={\rm{\Delta }}(\,\pm \,n\mp 1/2)$ and the linewidths of the modes are the same ${g}_{n}=g$ (where $T(0)=2\pi /{\rm{\Delta }}$ and ${{\rm{\Delta }}}_{\pm 1}=0.5$), we obtain the following set of the spectroscopic data $\{{{\rm{\Delta }}}_{\pm 1}=\pm \,0.5,\,{{\rm{\Delta }}}_{\pm 2}=\pm \,1.5,\,{g}_{\pm 1}=0.318,\,{g}_{\pm 2}=0.318\}$ (partial optimization, see (6)). After the complete optimization according (5) suppressing the negative spectral dispersion, for the same values $T(0)=2\pi /{\rm{\Delta }}$ and ${{\rm{\Delta }}}_{\pm 1}=0.5$ we obtain the following topological structure of optimal parameters for frequency detuning and linewidths: $\{{{\rm{\Delta }}}_{\pm 1}=\pm \,0.5,\,{{\rm{\Delta }}}_{\pm 2}=\pm \,1.92,\,{g}_{\pm 1}=0.318,\,{g}_{\pm 2}=1.09\}$ for four-particle system and $\{{{\rm{\Delta }}}_{\pm 1}=\pm \,0.5,\,{{\rm{\Delta }}}_{\pm 2}=\pm \,1.4,\,{{\rm{\Delta }}}_{\pm 3}=\pm \,3.0,\,{g}_{\pm 1}=0.32,\,{g}_{\pm 2}=0.24,\,{g}_{\pm 3}=1.6\}$ for six-particle system.

**Figure 3:**
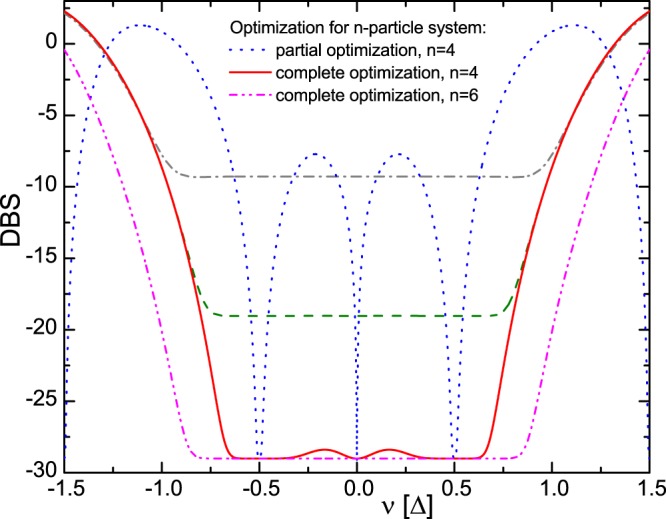
Spectral error of TF in the log scale $DBS(\nu )=10\,{\mathrm{log}}_{10}\,(\delta {S}^{2}(\nu ))$ for the four-particle system: red (solid) line – the complete parameter optimization and ${\gamma }_{n}\sim {10}^{-4}$, blue (dot) – the partial optimization and ${\gamma }_{n}\sim {10}^{-4}$, green (dash) – the complete optimization and ${\gamma }_{n}\sim {10}^{-3}$, gray (dash-dot) – the complete optimization and ${\gamma }_{n}\sim {10}^{-2}$; and for the six-particle system: magenta (dash-dot-dot) line – the complete optimization and ${\gamma }_{n}\sim {10}^{-4}$.

It is seen from the results of the numerical calculation of Eq. (2) given in Fig. 3, the comparison of the initial and optimized variants with allowance for internal losses ${\gamma }_{n}\sim {10}^{-4}$ achievable, e.g., upon using superconducting microwave resonators^34^ shows clearly the considerable improvement of spectral properties of the optimized variant (${\gamma }_{n}\sim {10}^{-4}$ corresponds to the quality factor $Q=5\cdot {10}^{6}$ of superconducting resonators for ${\rm{\Delta }}=3\cdot {10}^{7}$ and $\omega =3\cdot {10}^{10}$). Namely, in the second case the spectral quality of QM weakly depends of the frequency in the spectral interval from −$0.6{\rm{\Delta }}$ to $0.6{\rm{\Delta }}$ and $\delta {S}^{2}(\nu )\sim {10}^{-3}$ at ${\gamma }_{n}={10}^{-4}$. Thus, the optimization of parameters $\{{g}_{n},{{\rm{\Delta }}}_{n}\}$ makes it possible to create the almost ideal quantum interface in this frequency region with the QE: $\eta (\nu )\cong 0.999$. As it is seen in Fig. 3, it is possible only upon using the controlled multifrequency system. For comparison, we note that the AFC protocol on the atomic ensemble in the optical resonator with four resonance lines was recently implemented experimentally^12^, where the authors achieved the QE of 58% record for the AFC protocol. The result obtained in the work^12^ can be considerably improved in the proposed approach due to the optimization of the parameters related to the individual spectral lines.

Numerical simulation shows that the spectral behavior of high performance QM (spectral error *δS*^2^, respectively) is quite similar for ${N}_{0}=4$, ${N}_{0}=6$ and for larger number of mini-resonators (see Fig. 3). Herein, a larger number N can improve the spectral QEF and lead to the broader QM spectral width ∝${N}_{0}{\rm{\Delta }}$. However it should be necessary to find optimal parameters of all the absorbers due to the quite strong interaction in common resonator. Herein, the subsequent reversible transfer of the light field stored in the mini-resonators to the long-lived electron-nuclear spin system^19^ (for example in the rare-earth ions^35^) could provide on demand retrieval of the signal light.

## Discussion

We found that the merging of the eigenfrequencies (ST transition) can be observed in the discrete system of absorbers (atoms, mini-resonators etc.) interacting with common broadband cavity mode connected with the external waveguide. The considered QM is a linear device with respect to the input fields in accordance with the used linear system of Eq. (1) (i.e. the device works for arbitrary number of photons in the input light field), but the *E*_*n*_ eigenfrequency distribution depends nonlinearly on the controlled parameters of the studied system. A merging of the eigenfrequencies is an indicator of the range of optimal parameters near which the efficient quantum storage is achieved.

It was observed that the growth of the interaction constants *g*_*n*_ increases the QM spectral width for relatively weak interaction of the absorbers with the common mode, while considerable shifting and convergence of the central resonant lines and eigenfrequencies occur for larger *g*_*n*_. The detailed algebraic analysis of the observed line merging effects indicated to the presence of ST transition for the eigenfrequencies in this area of the spectroscopic parameters. Herein, the merging area of the two central lines determines the optimal value of the coupling constants *g*_*n*_ where the QM spectral width reaches its maximum and provides high QEF.

It is worth noting that control of eigenfrequencies topology is an important and necessary tool in the modern theory of broadband filters^30^, which we naturally extend here to the area of broadband QM. The proposed ST method for controlling the basic parameters of QM is universal for discrete multiparticle systems, where the number of controlled spectral parameters is finite and is implemented in practice. The used algebraic approach^29,31^ allows for analytically analyzing and optimizing all the physical parameters of the considered system (5) and also gives an exhaustive answer to the fundamental question of how to construct a superefficient QM corresponding to the theoretical limit.

In optics the proposed approach can be implemented in integral optical schemes containing systems of mini-resonators connected with a nanofiber^36,37^, where it is possible to control the frequencies of individual mini-resonators as well as its coupling with nanofibers^38^, and the usage of several atoms with tunable frequencies being in the common cavity is also possible. Superconducting resonators connected with planar waveguides^34^ is the most technological in the microwave frequency range. The developed ST approach of the multiparametric optimization of the QM opened the practical possibility of creating broadband high performance quantum interface with the non-destructive control^39,40^ consisting of a small countable number of resonance absorbers.

The spectral errors of quantum interface operation can be decreased to the extremely small values $\delta {S}^{2}(\nu )\sim {10}^{-3}$ that meets the technological requirements to QMs and its integration into quantum communication lines and quantum computer schemes. It is significant that the super-high quantum efficiency of more than 99.9% can be realized on the basis of current technologies and we have already conducted the first proof-of-principle experiments in this direction^18^.
